# Oxaliplatin(IV) Prodrugs Functionalized with Gemcitabine and Capecitabine Induce Blockage of Colorectal Cancer Cell Growth—An Investigation of the Activation Mechanism and Their Nanoformulation

**DOI:** 10.3390/pharmaceutics16020278

**Published:** 2024-02-16

**Authors:** Carlo Marotta, Damiano Cirri, Ioannis Kanavos, Luisa Ronga, Ryszard Lobinski, Tiziana Funaioli, Chiara Giacomelli, Elisabetta Barresi, Maria Letizia Trincavelli, Tiziano Marzo, Alessandro Pratesi

**Affiliations:** 1 Department of Chemistry and Industrial Chemistry, University of Pisa, 56124 Pisa, Italy; carlo.marotta@phd.unipi.it (C.M.); tiziana.funaioli@unipi.it (T.F.); 2 Institute of Analytical and Physical Chemistry for the Environment and Materials (IPREM-UMR 5254), Pau University, E2S UPPA, CNRS, 64053 Pau, France; i.kanavos@univ-pau.fr (I.K.); luisa.ronga@univ-pau.fr (L.R.); ryszard.lobinski@univ-pau.fr (R.L.); 3 Department of Pharmacy, University of Pisa, 56126 Pisa, Italy; chiara.giacomelli@unipi.it (C.G.); elisabetta.barresi@unipi.it (E.B.); maria.trincavelli@unipi.it (M.L.T.); tiziano.marzo@unipi.it (T.M.)

**Keywords:** Pt(IV)-based complexes, gemcitabine, capecitabine, PLGA nanoparticles, anticancer drugs, colorectal cancer

## Abstract

The use of platinum-based anticancer drugs, such as cisplatin, oxaliplatin, and carboplatin, is a common frontline option in cancer management, but they have debilitating side effects and can lead to drug resistance. Combination therapy with other chemotherapeutic agents, such as capecitabine and gemcitabine, has been explored. One approach to overcome these limitations is the modification of traditional Pt(II) drugs to obtain new molecules with an improved pharmacological profile, such as Pt(IV) prodrugs. The design, synthesis, and characterization of two novel Pt(IV) prodrugs based on oxaliplatin bearing the anticancer drugs gemcitabine or capecitabine in the axial positions have been reported. These complexes were able to dissociate into their constituents to promote cell death and induce apoptosis and cell cycle blockade in a representative colorectal cancer cell model. Specifically, the complex bearing gemcitabine resulted in being the most active on the HCT116 colorectal cancer cell line with an IC_50_ value of 0.49 ± 0.04. A pilot study on the encapsulation of these complexes in biocompatible PLGA-PEG nanoparticles is also included to confirm the retention of the pharmacological properties and cellular drug uptake, opening up to the possible delivery of the studied complexes through their nanoformulation.

## 1. Introduction

**Figure 1:**
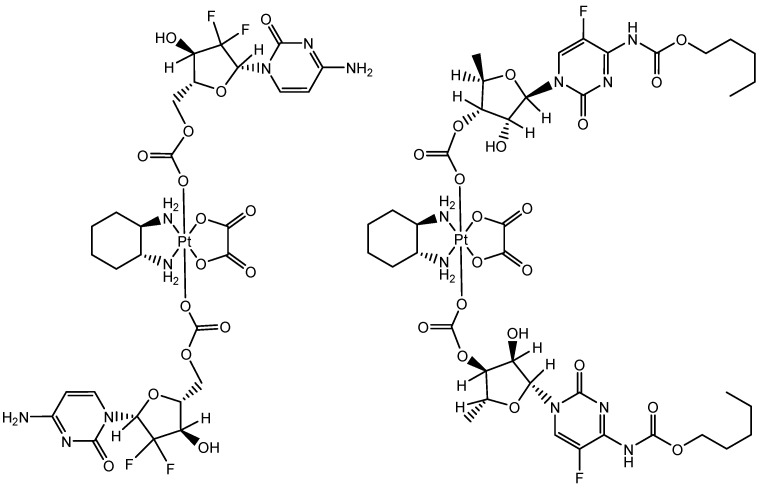
Chemical structure of oxaliplatin(IV)(Gem)_2_ (left, PTG hereafter) and oxaliplatin(IV)(Cape)_2_ (right, PTC hereafter).

Cancer is among the most common cause of death globally, and platinum-based anticancer drugs often represent frontline options in its management. Indeed, since the serendipitous discovery of cisplatin, these drugs have attracted growing attention due to their outstanding antiproliferative properties [1,2,3]. As a matter of fact, after cisplatin received FDA approval in the late 1970s, intensive research led to the discovery of and consequent clinical approval for cancer treatment of two other Pt(II) drugs (i.e., oxaliplatin and carboplatin) [4,5,6,7,8,9]. Nowadays, decades after the implementation of Pt compounds in the first clinical regimens, they still represent valuable weapons against tumor diseases. However, despite the undeniable clinical results, many drawbacks have emerged during the years of their medical use [10,11]. First of all, these drugs show debilitating side effects (such as severe vomiting, ototoxicity, nephrotoxicity, loss of appetite, and intense weakness) that in some cases force interruption of the clinical treatment [12,13,14,15,16,17,18]. Moreover, despite their initial effectiveness, it is not uncommon for cancer cells to develop resistance [12,15,16,17,18,19,20,21,22,23,24,25,26,27,28]. In order to overcome these drawbacks, many approaches have been explored over the years. Among them, the development of innovative platinum-based complexes is suitable and could be even improved through the combination of these drugs on different anticancer agents [29,30,31,32,33,34]. Indeed, combining different chemotherapeutic agents could offer many advantages, such as the possibility to target more pathways while reducing both the toxicity of the treatment and the insurgence of drug resistance [35]. In this context, the simultaneous administration of Pt(II) drugs with other organic chemotherapeutic agents has been intensively studied. For instance, the administration of oxaliplatin with 5-fluorouracil and folinic acid [6,36,37,38], with capecitabine [37,39,40], and with gemcitabine [41,42] led to the approval of specific clinical protocols to treat colorectal cancer, namely FOLFOX, CAPOX, and GEMOX, respectively. However, despite these approaches showing some advantages compared with single drug administration, they are not resolutive, and further research is needed. For this reason, other strategies have been taken into account, one being the modification of traditional Pt(II) drugs to obtain new molecules with ameliorated pharmacological profiles. In this context, Pt(IV) prodrugs have recently gained growing interest. Indeed, octahedral Pt(IV) complexes are usually more kinetically inert than their Pt(II) counterparts [26,43,44,45] and, therefore, they undergo fewer off-target reactions during their circulation in the body [26,43,45,46]. Furthermore, being prodrugs, they need to be activated to exert their anticancer activity. Their activation takes place after the cellular uptake process, preferentially in the hypoxic and reducing environment of the tumor, where they are reduced to the corresponding Pt(II) compounds with concomitant release of the two axial ligands [19,26,32,43,47,48,49,50,51,52,53,54,55,56]. This mechanism of activation is one of the reasons behind the interest in these complexes. Indeed, the axial position of these complexes can be conveniently functionalized with bioactive molecules which, after release in the cell, can potentially exert their anticancer activity alongside the Pt(II)-based drug, thus enhancing the overall pharmacological action [57,58,59,60]. Bearing this in mind, the rationale behind this work is to combine in a single Pt(IV) prodrug molecule two anticancer entities that were proven to be effective when administered together in combination therapy. Among the Pt(II) drugs, we selected oxaliplatin, and after oxidation to its Pt(IV) counterpart, we functionalized its axial positions with capecitabine or gemcitabine. Indeed, as aforementioned, oxaliplatin is already employed in the clinical protocols known as CAPOX [61,62] and GEMOX [41,42]. In turn, both capecitabine and gemcitabine are prodrugs. Capecitabine is a prodrug that belongs to the class of fluoropyrimidines. It is selectively tumor-activated to its active form, namely the antimetabolite 5-fluorouracil (5-FU), by thymidine phosphorylase [63]. Basically, from a mechanistic point of view, the latter acts through the inhibition of thymidylate synthase and the incorporation of its metabolites into RNA and DNA, causing the blockade of genetic material replication. Indeed, 5-FU is a pyrimidine analogue that undergoes misincorporation into both RNA and DNA in place of uracil or thymine. This event implies the triggering of interference with the biosynthesis and function of nucleic acids, determining the induction of apoptosis [63,64,65]. On the other hand, gemcitabine is a difluorinated uridine derivative and is biologically activated through intracellular phosphorylation by deoxycytidine kinase, which converts this prodrug into its mono-phosphate analogue, which is subsequently transformed into the active form of the drug (gemcitabine di- and triphosphate) [66,67]. The main mechanism of action of this drug involves the inhibition of DNA synthesis, which leads to cell death [67,68]. Indeed, after gemcitabine triphosphate has been incorporated into the DNA in place of deoxycytidine triphosphate, the DNA polymerase is able to insert only another nucleotide into the chain. Subsequently, termination of the chain elongation takes place, leading to DNA fragmentation and cell death [66,67]. Here, we report the design, synthesis, and characterization of two novel Pt(IV) prodrugs based on oxaliplatin bearing the anticancer drug gemcitabine or capecitabine in the axial positions (Figure 1). By combining these chemotherapeutic agents with oxaliplatin in a single molecule, we might be able to overcome some of the limitations of their combination therapy. For instance, the side effects might be reduced because, according to the mechanism of activation of Pt(IV) prodrugs, the biologically active moieties will be released only after reduction inside the cancer cell [54,69]. Furthermore, since different pathways are simultaneously targeted by the two drugs combined in a single molecule, once released in the cancer cell, the drug resistance mechanisms that are observed in single-drug treatment regimens might be overcome. Accordingly, this approach is currently attracting growing attention [70,71], representing a valid strategy for the synthesis of new anticancer prodrugs.

The proposed compounds have been studied through cyclic voltammetry and mass spectrometry (MS), and their antiproliferative capabilities were elucidated on a colorectal cancer model (HCT116). More precisely, the anticancer properties of these platinum(IV) compounds have been evaluated in comparison to parent compounds oxaliplatin, capecitabine, and gemcitabine. The biological experiments have been performed both with standard protocols and after the encapsulation of the compounds in biocompatible PLGA-PEG nanoparticles to assess the retention of the properties of PTC and PTG upon encapsulation in biocompatible nanoplatform, opening up the avenue to further development and evaluation of the complexes. Indeed, the loading of nanovectors with anticancer molecules is widely recognized as an exploitable strategy to both lowering side effects (because of the shielding of the active drugs from off-target reactions) and enhancing the targeting towards cancer (for instance, by decoration of the nanoparticles’ external surface with chemical or biological entities capable of allowing the selective recognition of the nanoparticle by the cancer cells) [72].

## 2. Materials and Methods

All solvents and chemical reagents were purchased either from Sigma-Aldrich (St. Louis, MO, USA) and Fluorochem (Glossop, UK).

### 2.1. Synthesis

#### 2.1.1. Building Block Preparation

Oxaliplatin was prepared through classical synthetic procedures and characterized through ^1^HNMR and elemental analysis [73]. The proton spectrum was fully consistent with those reported in the literature [74]. Oxaliplatin(IV)(OH)_2_ was prepared through a modified reported oxidation procedure [75]. More precisely, 300 mg of oxaliplatin was suspended in 10 mL of deionized water. The mixture was stirred at room temperature, and 2 mL of 30% hydrogen peroxide solution was added. The suspension was stirred overnight, and then most of the solvent was removed through a rotary evaporator. The milky mixture was diluted with ethanol, and the formed suspension was filtered on a glass membrane to recover the crude product. The white powder was washed with ethanol and diethyl ether and dried under reduced pressure. Finally, 280 mg of the pure product was recovered (yield 84%). The product was characterized through ^1^HNMR and elemental analysis. ^1^HNMR (400 MHz, D_2_O) δ: 2.83 (m; 2H), 2.72 (m; 2H), 1.60 (m; 4H), 1.25 (m; 2H). Elemental analysis: calc. C 22.28%, H 3.74%, N 6.50%; exp. C 21.76%, H 3.52%, N 6.21%.

Di-Boc-gemcitabine was prepared and characterized as reported in a previous paper [76]. MSC-di-Boc-gemcitabine was prepared as reported in the literature [77], where MSC is mono succinimidyl carbonate. Oxaliplatin(IV)(MSC)_2_ was obtained in a quantitative yield following the procedure reported in the literature [74].

#### 2.1.2. PTC Preparation

Oxaliplatin(IV)(Cape)_2_ (PTC) was prepared as follows: 170 mg (0.238 mmol; 1.00 eq.) of oxaliplatin(IV)(MSC)_2_ was solubilized in 2 mL of anhydrous DMSO together with 214 mg (0.596 mmol; 2.50 eq.) of capecitabine and 29 mg (0.24 mmol; 1.0 eq.) of 4-(dimethylamino)pyridine. The mixture was stirred overnight, and then diethyl ether was added until the formation of an insoluble oil. The supernatant was removed, and the oil was solubilized in 0.5 mL of methanol. The crude product was recovered through precipitation with diethyl ether. The product (91 mg; yield 32%) was obtained through purification on an Isolera system for flash column chromatography on reverse-phase C18 silica (Biotage, Uppsala, Sweden). The elution was performed through an elution gradient with methanol and water.

^1^HNMR (400 MHz, DMSO-d_6_) δ: 8.55 (m; 2H; carbamate group), 8.03 (m; 2H; aromatic), 7.07 (m; 4H; amino group), 3.58–0.84 (unattributable multiplets). Elemental analysis: calc. C 39.97%, H 4.70%, N 9.32%; exp. C 39.48%, H 5.12%, N 9.73%.

#### 2.1.3. PTG Preparation

Oxaliplatin(IV)(Gem)_2_ (PTG) was prepared as follows: 455 mg (0.770 mmol; 2.50 eq.) of MSC-di-Boc-gemcitabine and 140 mg (0.320 mmol; 1.00 eq.) of oxaliplatin(IV)(OH)_2_ were solubilized in 2 mL of DMSO. The mixture was stirred overnight, and then diethyl ether was added until the formation of an insoluble oil. The supernatant was removed, and the crude product was solubilized in 2 mL of dichloromethane. Subsequently, 1 mL of TFA was added, and the solution was stirred at room temperature for 45 min. The solution was then dried through a rotary evaporator, and the residual oil was solubilized with about 0.5 mL of methanol. The crude product was recovered through precipitation with diethyl ether. The product (184 mg; yield 56%) was obtained through purification on an Isolera system for flash column chromatography on reverse-phase C18 silica (Biotage, Uppsala, Sweden). The elution was performed through an elution gradient with methanol and water.

^1^HNMR (400 MHz, DMSO-d_6_) δ: 8.43 (m; 4H; amino group), 7.84 (m; 4H; amino group), 7.48 (m; 2H; aromatic), 7.38 (m; 2H; aromatic), 6.12 (b; 2H; sugar), 5.75 (m; 2H; sugar), 4.37–3.82 (m; 6H; sugar), 2.05 (m; 2H; DACH), 1.47 (m; 4H; DACH), 1.08 (m; 2H; DACH). Elemental analysis: calc. C 33.31%, H 3.39%, N 11.10%; exp. C 32.87%, H 2.91%, N 10.82%.

### 2.2. Nanoparticles Preparation

Nanoparticle formulations employed for biological tests were prepared with PLGA-PEG-COOH copolymer. The polymeric matrix and the nanoparticles were prepared and characterized following the method described in a previous paper with some modifications, as described below. This method allows for nanoparticles with a dimension of 65 nm to be obtained [72]. The final concentration of PTG loaded in the nanoparticle was executed through ICP-AES, as previously described [72].

#### 2.2.1. Empty Nanoparticles Preparation

Empty nanoparticles were prepared by solubilizing 28.8 mg of PLGA-PEG-COOH polymeric matrix in 0.5 mL of dimethyl sulfoxide and 4.5 mL of acetonitrile. The solution was added dropwise in 20 mL of demineralized water under stirring. The final mixture was evaporated for 30 min at r.t. through a rotary evaporator to remove the acetonitrile. After evaporation, 19.2 mL of suspension was recovered.

#### 2.2.2. Oxaliplatin/Gemcitabine-Loaded Nanoparticles

Nanoparticles loaded with a 2:1 molar ratio of gemcitabine and oxaliplatin were prepared by solubilizing 2.54 mg of oxaliplatin in 0.5 mL of dimethyl sulfoxide. Subsequently, we solubilized 28.5 mg of PLGA-PEG-COOH polymeric matrix and 3.5 mg of gemcitabine in 4.5 mL of acetonitrile. The two solutions were mixed, and the resulting mixture was added dropwise in 20 mL of demineralized water under stirring. The final mixture was evaporated for 30 min at r.t. through a rotary evaporator to remove the acetonitrile. After evaporation, 18 mL of suspension was recovered.

#### 2.2.3. PTG-Loaded Nanoparticles

Nanoparticles loaded with PTG were prepared by solubilizing 6.5 mg of PTG in 0.5 mL of dimethyl sulfoxide. Subsequently, we solubilized 28.8 mg of PLGA-PEG-COOH polymeric matrix in 4.5 mL of acetonitrile. The two solutions were mixed, and the resulting mixture was added dropwise in 20 mL of demineralized water under stirring. The final mixture was evaporated for 30 min at r.t. through a rotary evaporator to remove the acetonitrile. After evaporation, 14 mL of suspension was recovered.

### 2.3. NMR Spectroscopy

NMR spectroscopy. All NMR spectra were acquired on a Jeol 400YH spectrometer (Akishima, Tokyo, Japan). All spectra were recorded at room temperature (25 ± 2 °C) in solvents with a deuteration degree of 99.8% and calibrated on solvent residual signals. All deuterated solvents were purchased from Deutero (https://www.deutero.de/ accessed on 9 February 2024).

### 2.4. Mass Spectrometry Analysis

Sample preparation. A stock solution of ODN2 1 mM was prepared by dissolving the sample in ultrapure water. Ammonium acetate solution (2 mM, pH 7.0) was prepared by weighing ammonium acetate and dissolving it in ultrapure water, and pH adjustment was carried out with acetic acid and ammonia commercial solutions (35% NH_3_). Stock solutions of oxaliplatin 10 mM, PTG 10 mM, flavin mononucleotide (FMN) 1 mM, and nicotinamide adenine dinucleotide (NADH) 100 mM were prepared by solubilizing the solid compounds in ultrapure water. Aliquots of ODN2 stock solution were diluted with 2 mM ammonium acetate solution (pH 7.0) to 0.1 mM final oligonucleotide. For incubation with Pt compounds, three different mixtures were prepared by being added to this ODN2 solution: (i) oxaliplatin (1:3 ODN2-to-oxaliplatin ratio) or (ii) PTG (1:3 ODN2-to-PTG ratio) or (iii) PTG (1:3 ODN2-to-PTG ratio) in the presence of NADH (4:1 NADH-to-PTG ratio) and FMN (1:4 FMN-to-PTG ratio). The mixtures were explored over different incubation times (24 and 48 h) at 37 °C. After incubation, these three solutions were sampled and diluted to a final ODN2 concentration of 1–6 μM using H_2_O and 50% AcN solution. Electrospray–MS. Flow injection analysis (FIA) was performed using Dionex Ultimate 3000 series UHPLC from Thermo Fisher Scientific (Waltham, MA, USA) coupled to an Orbitrap Q-Exactive Plus mass spectrometer from Thermo Fisher Scientific. The mobile phases were A: H_2_O and B: acetonitrile. The flow rate used in FIA-MS experiments was 1 mL min^−1^ over 3 min with 50% B. Injection volumes of 10 μL were used. Ionization was performed using an electrospray ion source operating in negative ion mode with a capillary voltage of 2.7 kV and capillary temperature of 320 °C. Sheath gas, auxiliary gas, and sweep gas flow rate were set at 15, 5, and 1 (arbitrary units), respectively. The auxiliary gas temperature was set at 0 °C. The deconvolution of the masses observed in the spectra was performed by Thermo Fisher Scientific Xcalibur FreeStyle 4.5 software (Waltham, MA, USA).

### 2.5. Cyclic Voltammetry

Electrochemical experiments were performed with a PalmSens4 instrument equipped with PSTrace5 electrochemical suite and were recorded at room temperature in an aqueous solution. Phosphate buffer (PB) solution (Na_2_HPO_4_/KH_2_PO_4_, Σc_(PO4)_ = 50 mM, pH = 7.0) was prepared in ultrapure H_2_O and used as an aqueous supporting electrolyte. Cyclic voltammetry was performed experimentally in a three-electrode cell: the working electrode was a Teflon encapsulated carbon-glassy stick, the counter electrode was a platinum gauze, and the reference electrode was a leakless miniature Ag/AgCl/KCl electrode (eDAQ). Prior to measurements, the glassy carbon working electrode was polished according to the following procedure: manual rubbing with 0.3 μm Al_2_O_3_ slurry in water (eDAQ) for 2 min, then sonication in ultrapure water for 10 min, manual rubbing with 0.05 μm Al_2_O_3_ slurry in water (eDAQ) for 2 min, and then sonication in ultrapure water for 10 min. The supporting electrolyte solution was introduced into the cell and degassed through bubbling argon gas. The working electrode potential was cycled several times between the cathodic and anodic limits to determine the solvent window. The analyte was then introduced into the cell, and the voltammogram was recorded (0.1 V s^−1^) under a blanket of argon.

### 2.6. Biological Experiments

Cell culture. HCT116 cells (Human colorectal carcinoma cells) were kindly provided by Prof. Tania Gamberi (Department of Experimental and Clinical Biomedical Sciences “Mario Serio”, University of Florence). Cells were maintained in DMEM-F12 (Corning, Corning, NY, USA) supplemented with 10% FBS (Corning), 2 mM L-glutamine, 100 U/mL penicillin, and 100 μg/mL streptomycin at 37 °C in a humidified 5% CO_2_ atmosphere. Cell viability assay. Cells were seeded in 96-well microplates (3500 cells/well) and treated with different concentrations of the compounds, i.e., PTG, PTC, PTG-loaded nanoparticles (NPTG), nanoparticles loaded with oxaliplatin and gemcitabine (NP Pt + Gem), capecitabine, gemcitabine, and oxaliplatin (ranging from 10 nM up to 2 mM). The final DMSO concentration was 1%. Then, cell viability was determined using an MTS assay (CellTiter 96 Aqueous One Solution Cell Proliferation Assay kit; Promega, Madison, WI, USA) according to the manufacturer’s instructions. The absorbance values at 490 nm were measured with the Victor Wollac 2 multimode plate reader (Perkin Elmer, Whaltham, MA, USA). Quantification of cell cycle and apoptosis. HCT116 cells were seeded in 6-well microplates (90,000 cells/well). After 24 h, cells were treated with DMSO (CTRL) or PTG, PTC, gemcitabine, capecitabine, and oxaliplatin for 48 h. The evaluation of the cell cycle was performed using the Muse Cell Cycle Assay Kit (MCH100106) with the Muse Cell Analyzer (Merck KGaA, Darmstadt, Germany), as previously described [74]. After treatment in the same conditions, the number of apoptotic cells was evaluated using the Muse Annexin V and Dead Cell Assay Kit (MCH100105) with the Muse Cell Analyzer instrument [78]. Statistical analysis. Data were analyzed using the Graph-Pad Prism 6.0 (GraphPad Software Inc., San Diego, CA, USA). The IC_50_ values were calculated using the “non-linear fit log(inhibitor) vs. normalized response—Variable slope”.

## 3. Results and Discussion

### 3.1. PTC and PTG Synthesis

**Scheme 1:**
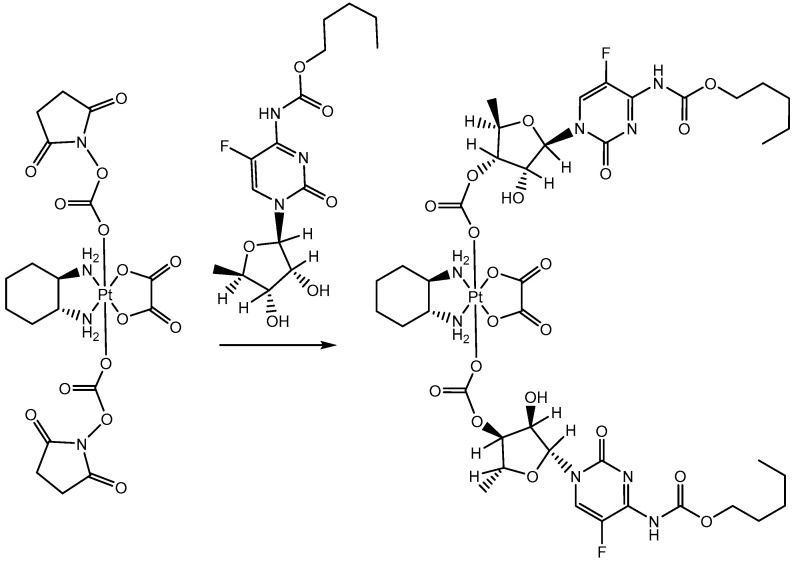
Synthetic route for PTC.

**Scheme 2:**
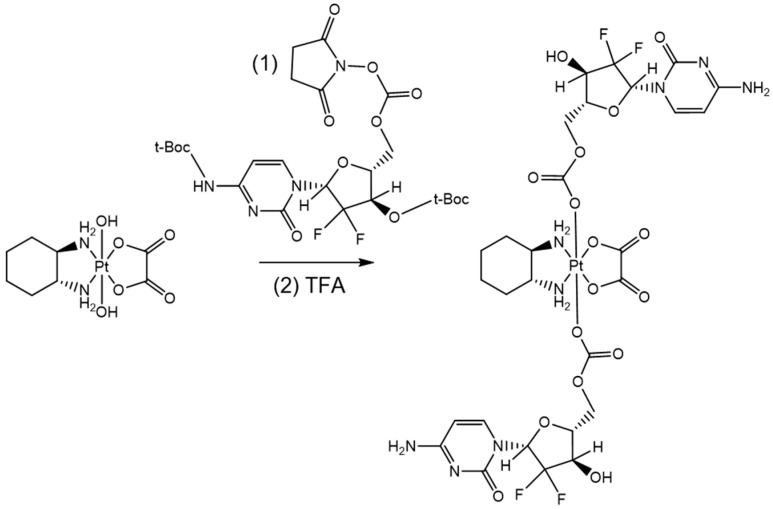
Synthetic route for PTG.

The synthesis of both compounds started from oxaliplatin synthesis [73] and its subsequent oxidation to the corresponding Pt(IV) analogue, i.e., oxaliplatin(IV)(OH)_2_. More precisely, oxaliplatin(IV)(OH)_2_ was prepared through a classical oxidation procedure carried out by exploiting a concentrated solution of hydrogen peroxide [75]. Oxaliplatin(IV)(Cape)_2_ (PTC) was prepared as shown in Scheme 1: First of all, we synthesized the succinimidyl carbonate diester of oxaliplatin(IV)(OH)_2_, i.e., oxaliplatin(IV)(MSC)_2_. The reaction was carried out at room temperature in DMF with an excess of disuccinimidyl carbonate with respect to oxaliplatin(IV)(OH)_2_. The intermediate was precipitated after 2 h with acetonitrile, filtered out with an Hirsch funnel, and stored at −20 °C. PTC was then prepared by reacting overnight capecitabine with the obtained oxaliplatin(IV)(MSC)_2_ in DMSO and 4-(dimethylamino)pyridine as a catalyst. Conversely, for obtaining PTG (Scheme 2), we firstly protected the gemcitabine with two t-Boc moieties (to avoid the interference of 3′-oxygen and aminic nitrogen) [76] and then prepared the active monosuccinimidyl ester, i.e., the di-(t-Boc)-gemcitabine(MSC) [77]. The main coupling among oxaliplatin(IV)(OH)_2_ and di-(t-Boc)-gemcitabine(MSC) was conducted in DMSO and afforded the di-(t-Boc)-oxaliplatin(IV)(Gem)_2_ intermediate. Finally, this latter compound was deprotected with a mixture of trifluoroacetic acid (TFA) and dichloromethane.

### 3.2. Effects of PTC and PTG on Cell Growth

**Figure 2:**
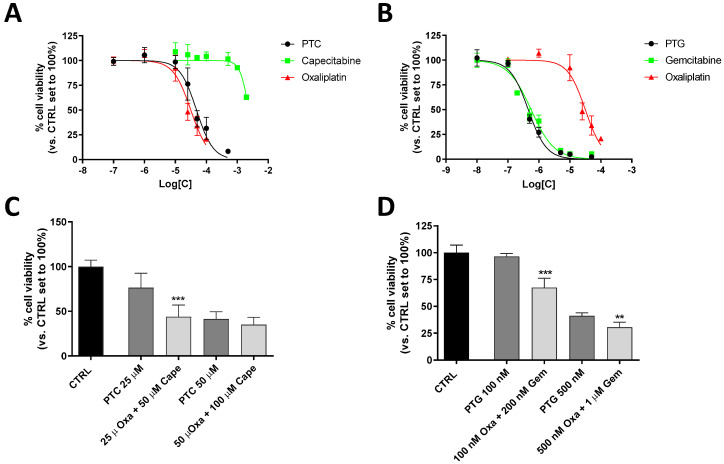
Effects of PTG and PTC on HCT116 cell viability. The cells were treated with the reported concentrations for 72 h. (**A**,**B**) Dose-response curves of PTC, PTG, capecitabine, gemcitabine, and oxaliplatin. (**C**,**D**) Effects on cell viability of PTC, PTG, and the combined treatments (as reported in the graph). At the end of the treatments, cell viability was measured using the MTS assay. The data were expressed as a percentage with respect to the untreated cells, which were set to 100%, and they were presented as the mean values ± SD of three independent experiments each performed in duplicate. The statistical significance of the differences was determined with a one-way ANOVA with Bonferroni’s post-test: ** *p* < 0.01, *** *p* < 0.001 vs. respective PTG and PTC treatment.

The growth inhibition activity of oxaliplatin in the human colon cancer cell line (HCT116) was previously reported [79,80]. Here, the effects of the new complexes, namely PTC and PTG, on the HCT116 cell line were investigated and compared to oxaliplatin, gemcitabine, and capecitabine administered as single agents or as oxaliplatin–gemcitabine–capecitabine combinations (Table 1, Figure 2). As expected, oxaliplatin treatment (100 nM–100 µM) for 72 h caused a significant decrease in proliferation, yielding an IC_50_ value of 29 ± 9 µM. Gemcitabine alone was able to decrease the HCT116 cell viability in a dose-dependent manner with an IC_50_ value of 0.53 ± 0.05 µM. Capecitabine treatment was able to slightly affect the HCT116 cell viability only at high concentrations (2 mM). In parallel, the effects of the two complexes PTC and PTG on HCT116 viability were evaluated. Both complexes were able to decrease the HCT116 viability to a different extent (IC_50_ = 50 ± 8 µM and 0.49 ± 0.04, respectively). Notably, the IC_50_ values of each complex reflected the inhibition capability of the most active component; specifically, PTC had comparable activity to oxaliplatin and PTG to gemcitabine (Figure 2A,B). To investigate if the activity of both complexes could derive from the additive effects of their components, two concentrations of each complex were chosen and compared to the effects induced by treatment with a combination of the corresponding amount of their components (Figure 2C,D). As evidence, the combination of capecitabine (50 µM) and oxaliplatin (25 µM) produced a significantly higher inhibition with respect to PTC (25 µM), even if the reduction became comparable when higher doses were evaluated, namely PTC 50 µM vs. oxaliplatin 50 µM + capecitabine 100 µM (Figure 2C). Similarly, the combination of gemcitabine and oxaliplatin produced a significantly higher inhibition with respect to PTG at both tested concentrations (Figure 2D). Altogether, these data highlighted that the complexes are able to release their axial ligands, together with the reduction product oxaliplatin, promoting HCT116 cell death. We can also speculate that these experiments pointed out the role of the kinetic of complexes’ disassembling in determining the overall pharmacological effects in comparison with the corresponding mixture of the free drugs (see Figure 2). Nevertheless, on the other hand, it should be noticed that the higher kinetic inertness of Pt(IV) compounds ensures a higher selectivity because the reductive activation step mainly occurs once the complexes enter cells.

### 3.3. Apoptosis Induction and Cell Cycle Blockade

**Figure 3:**
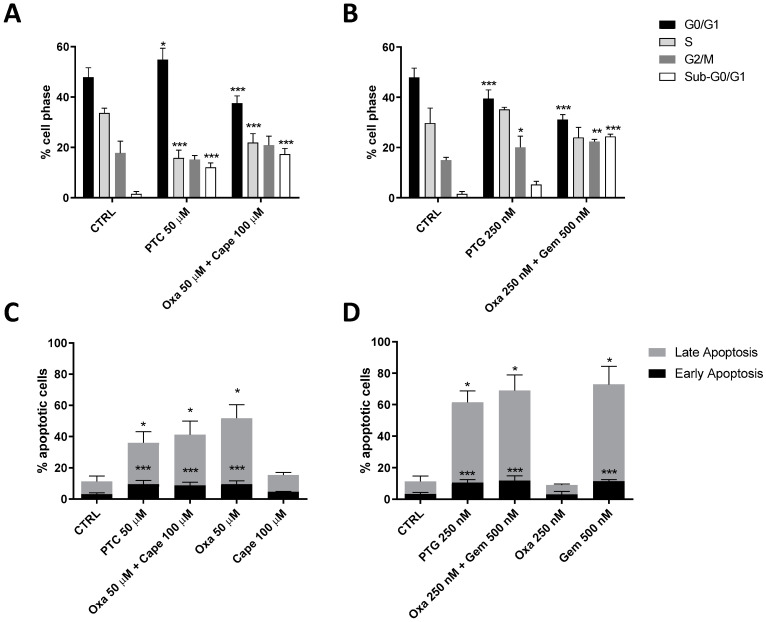
PTG and PTC trigger cell cycle disturbance and apoptosis in HCT116 cells. HCT116 cells were treated as reported for 48 h. After incubation time, (**A**,**B**) the cell cycle distribution or (**C**,**D**) apoptosis induction were evaluated. (**A**,**B**) Data are expressed as the percentage of cells in the different phases (sub-G0/G1, G0/G1, S, or G2/M) versus total cell number. Data represent the mean ± SD of three different experiments. The statistical significance of the differences was determined with a one-way ANOVA with Bonferroni’s post-test: * *p* < 0.05, ** *p* < 0.01, *** *p* < 0.001 vs. respective phase of the CTRL. (**C**,**D**) The phosphatidylserine externalization was quantified using Annexin V staining. The data are expressed as the percentage of apoptotic cells (early apoptotic in black, late apoptotic in grey) versus the total number of cells. Data represent the mean ± SD of three different experiments. The statistical significance of the differences was determined with a one-way ANOVA with Bonferroni’s post-test: * *p* < 0.05, ** *p* < 0.01, *** *p* < 0.001 vs. respective early or late apoptosis of the CTRL.

The decrease in cell viability can be the result of different processes [81]. Thus, to deeply investigate the mechanisms of action of the PTC and PTG complexes, their ability to induce apoptosis and block cell cycle progression was investigated by cytofluorimetric assays (Figure 3 and Figures S1–S3). Challenging HCT116 cells with IC_50_ values of PTC and PTG (50 µM and 500 nM, respectively) for 48 h induced a significant arrest of the cell cycle. Interestingly, the PTC caused a significant increase in the DNA content in G0/G1 phase and a concomitant decrease in the S phase cell number, highlighting its ability to induce a cell cycle blockade in G0/G1 (Figure 3A). Notably, the complex treatment caused a significant increase in sub-G0/G1 cells, reflecting the presence of apoptotic cells. This effect was more evident after the treatment with the mix of capecitabine (100 µM) and oxaliplatin (50 µM). The ability of capecitabine to induce a cell cycle arrest in G0/G1 phase in non-small-cell lung cancer cells has been reported [82]. Interestingly, the effects of its active metabolite 5-FU differ based on the type of cancer cells [83]. Accordingly, the ability of oxaliplatin to induce both G0/G1- and G2/M-phase arrest in HCT116 cells has also been reported [84,85]. Treatment with a 500 nM concentration of PTG caused a significant increase in sub-G0/G1 cells, producing a decrease in cells in all the cell cycle phases (See Figure S1). Thus, a lower concentration of PTG (250 nM) was used to highlight a specific effect on cell cycle blockade (Figure 3B). The results demonstrated that this complex was able to promote a cell cycle blockade in G2/M phase, causing a decrease in G0/G1 cells and an increase in G2/M cells. Similar effects were evidenced after treatment with a combination of gemcitabine and oxaliplatin. Notably, the sub-G0/G1 population was increased, especially in the gemcitabine and oxaliplatin combination treatment, highlighting apoptotic phenomena. Gemcitabine enters the cells, and after its phosphorylation, it is incorporated into replicating DNA strands, resulting in DNA damage and cell cycle blockade in different phases based on the treatment time and type of cancer cells [86]. Taken together, these results demonstrate the ability of both complexes to reduce cell viability through cell cycle perturbation, like the effects prompted by oxaliplatin, gemcitabine, or capecitabine alone. To better investigate the cellular effects of the complexes, we investigated the induction of apoptosis after 48 h of treatment (Figure 3C,D). PTC (50 µM) caused a significant phosphatidylserine externalization, both in the absence (early apoptosis) and presence of 7-aminoactinomycin D (7-AAD) binding to DNA (late apoptosis/death; Figure 3C). The oxaliplatin and capecitabine combination promoted apoptosis to a similar extent in accordance with the reduction in cell viability. Similarly, PTG (250 nM) and oxaliplatin–gemcitabine combination increased the number of early and late apoptotic cells (Figure 3D). Interestingly, the extent of apoptosis promoted by the PTC and PTG reflects the effect of oxaliplatin and gemcitabine alone, respectively, in accordance with the data obtained in the cell viability assay (Figure 2C,D). Despite the observed biological effects being mainly driven and attributable to the most active compounds produced through reduction (oxaliplatin in the case of PTC or in mixture with capecitabine and gemcitabine in the case of PTG or in the mixture with oxaliplatin), mechanistically, we can assume that the kinetic of Pt(IV) reduction impacts the overall anticancer effects. Finally, it should also be highlighted that the 1:2 ratio between Pt and the axial ligands forces the tested drugs’ concentration to selected doses to perhaps be different from the exact effective doses determined through IC_50_ determination on single drugs.

### 3.4. Oxaliplatin(IV)(Gem)_2_ Reactivity Studies

**Figure 4:**
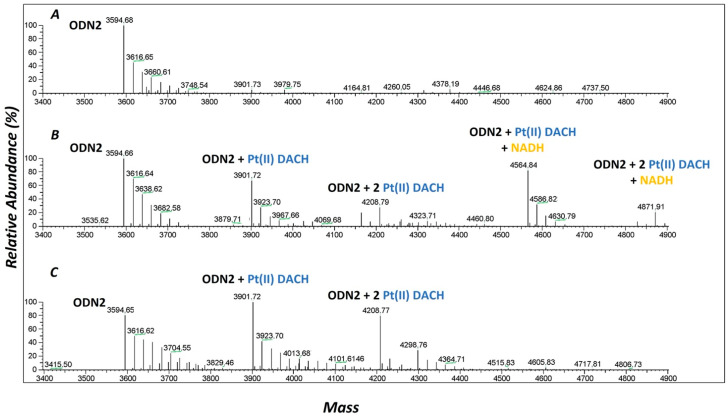
Deconvoluted ESI mass spectra of (**A**) ODN2 (10^−4^ M) incubated at 37 °C for 24 h with PTG (1:3 ODN2-to-PTG ratio) in 2 mM ammonium acetate solution pH = 7.0, (**B**) ODN2 (10^−4^ M) incubated at 37 °C for 24 h with PTG (1:3 ODN2-to-PTG ratio) in 2 mM ammonium acetate solution pH = 7.0 in the presence of NADH (4:1 NADH-to-PTG ratio), and FMN (1:4 FMN-to-Pt(IV)-Gem ratio), (**C**) ODN2 (10^−4^ M) incubated at 37 °C for 24 h with oxaliplatin (1:3 ODN2-to-oxaliplatin ratio) in 2 mM ammonium acetate solution pH = 7.0. Moreover, based on the relative intensity of mass signals of unreacted ODN2 and its Pt adducts, the metallation extent of ODN2 appeared to increase over a longer incubation time (48 h) with PTG (Figure S4).

Since PTG showed very promising results in the preliminary biological evaluation, it was subjected to further investigations. Firstly, the ability of PTG complex to dissociate in its constituents in cancer cells was investigated by high-resolution electrospray (ESI) MS. Accordingly, it was possible to assess as PTG was unreactive with a model oligonucleotide biomolecule until its reduction. This latter event, indeed, leads to the active Pt(II) analogue (oxaliplatin); in turn, this implies the concomitant release of the axial ligands. The ODN2 oligonucleotide (sequence: 5′-CTACGGTTTCAC-3′, molecular weight: 3596.36 Da) bearing the GG motif, a preferential binding site for Pt-containing molecules [87], was selected and reacted with PTG both in the presence or in the absence of an efficient reducing system based on flavin (FMN) and NADH, which is naturally present in the cellular environment [88,89,90,91,92]. As shown in Figure 4, only upon the incubation of ODN2 with PTG for 24 h, in the presence of FMN and NADH, was the formation of ODN2/Pt(II)-DACH mono- and bis-adducts detected (comparisons of experimental and theoretical isotopic patterns are shown in Figure S5 and Figure S6, respectively). Under these conditions, PTG complex induced the formation of the same type of ODN2/Pt(II) adducts observed by reacting ODN2 with oxaliplatin. This confirms the ability of this complex to undergo Pt(IV) reduction to oxaliplatin and the subsequent release of axial ligands (gemcitabine), promoted by the FMN/NADH reducing system. Interestingly, the NADH also participated in the formation of two additional Pt(II)-DACH mono- and bis-adducts with an extra NADH moiety, probably coordinating the model oligonucleotide (comparisons of experimental and theoretical isotopic patterns are shown in Figures S7 and S8).

### 3.5. Cyclic Voltammetry

**Figure 5:**
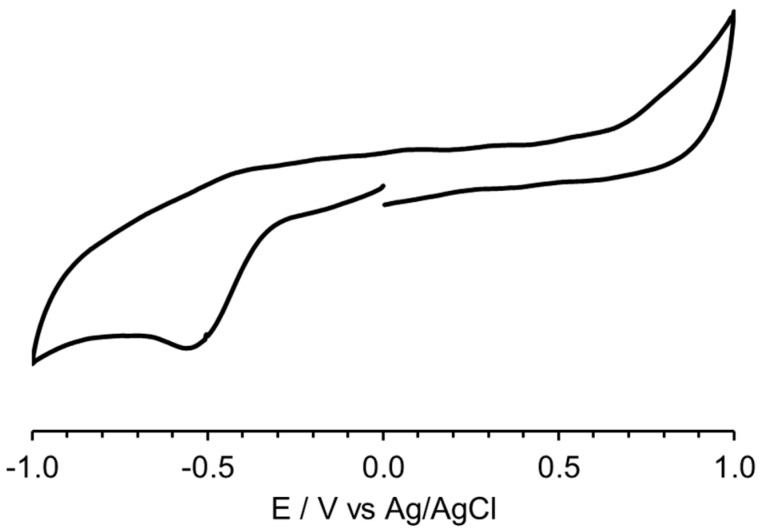
Cyclic voltammetry of PTG in 0.05 M phosphate buffer (pH 7.0) at a glassy carbon electrode. Scan rate: 0.1 V s^−1^.

The antitumor activity of Pt(IV) derivatives relies on the formation of active Pt(II) species through the so-called “reductive activation” mechanism [93]. The electrochemical behavior of PTG was studied by cyclic voltammetry in 0.05 M phosphate buffer (pH 7.0) at a glassy carbon electrode (Figure 5). PTG undergoes an irreversible reduction at Ec = −0.55 V vs. Ag/AgCl (−0.35 V vs. SHE), a potential value within the biologically relevant range of potentials that approximately covers the window −0.4 to +0.8 V vs. SHE (−0.6 to +0.6 V vs. Ag/AgCl) [94,95]. Also, the irreversible reduction waves indicate that PTG releases axial ligands upon reduction from Pt(IV) to Pt(II), with the concomitant switching from octahedral to square planar geometry [96].

### 3.6. Encapsulation of PTG in Nanoparticles

Although modifying the structure of the antitumor drugs is a valuable approach for the discovery of new anticancer drugs, an additional strategy to expand the pharmacological properties of compounds to improve the pharmacological effects—concomitantly leading to lower side effects—is the encapsulation of anticancer-active entities into biocompatible nanoparticles. Nowadays, several successful examples of drug encapsulation and delivery have been reported in the scientific literature [97,98,99,100,101]. On this basis, we also performed a pilot study based on the encapsulation of PTG in the core of PLGA-PEG nanoparticles, thus forming the aforementioned drug-delivery system. Moreover, since both PLGA and PEG polymers are biodegradable and biocompatible, they are particularly exploitable for biological applications [102,103,104]. Remarkably, these devices are also used to deliver compounds with low solubility in water. For instance, the water solubility of Pt(IV) complexes can sometimes be reduced with respect to their precursors when bulky organic moieties are tethered to their axial position. In order to make their administration possible, one solution consists of encapsulating these drugs in biocompatible nanoparticles [72,105]. Also, nano-formulations of the Pt(IV) complexes are useful for preventing unwanted reactions because they do not undergo early reduction or hydrolysis in the extracellular environment [60]. Experimentally, the preparation of PLGA-PEG drug-loaded nanoparticles has been already described in the literature [72]. Briefly, the PLGA-PEG copolymer is prepared through an amidation process among 11 kDa PLGA and 3.4 kDa NH_2_-PEG-COOH, while the loading step is described in detail in the experimental section. The final concentration for the mix oxaliplatin/gemcitabine and for PTG, determined through ICP-AES analysis, was 4.67 × 10^−4^ and 4.29 × 10^−4^ M, respectively.

### 3.7. Effects of PTG-Loaded Nanoparticles on Colon Cancer Cell Growth

**Figure 6:**
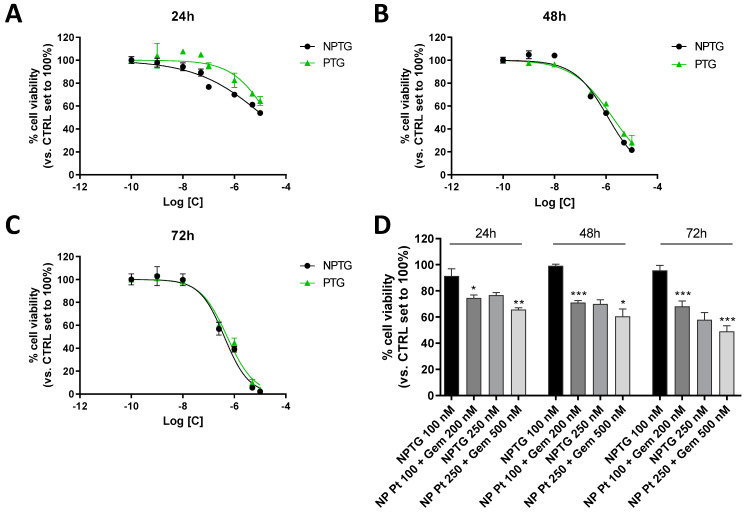
Effects of NPTG and NP Pt + Gem on HCT116 cell viability. The cells were treated with the reported concentrations for 24 (**A**), 48 (**B**), or 72 h (**C**). The dose-response curves are reported. (**D**) Effects of the NPTG and NP Pt + Gem on cell viability. At the end of the treatments, cell viability was measured using the MTS assay. The data were expressed as a percentage with respect to the untreated cells, which were set to 100%, and they were presented as the mean values ± SEM of three independent experiments each performed in duplicate. The statistical significance of the differences was determined with a one-way ANOVA with Bonferroni’s post-test: * *p* < 0.05, ** *p* < 0.01, *** *p* < 0.001 vs. respective NPTG treatment.

To demonstrate the maintained cytotoxic potential and the possible existence of different properties for PTG-loaded nanoparticles (NPTG), the reduction in HCT116 cell viability was evaluated at different time points (24–72 h) and compared to the free PTG (Figure 6, Table 2). Both the PTG and NPTG were able to significantly decrease the HCT116 viability at 24 h. Interestingly, the nanoparticle formulation demonstrated a lower IC_50_ value with respect to the complex alone (IC_50_ = 1.37 ± 0.19 µM and 2.3 ± 0.2 µM, respectively) (Figure 6A). The same trend was evidenced after 48 h of treatment (IC_50_ = 1.21 ± 0.03 µM and 1.7 ± 0.2 µM, NPTG and PTG, respectively) (Figure 6B). The difference in the cytotoxic effect was completely lost after 72 h of treatment (IC_50_ = 0.44 ± 0.08 µM and 0.50 ± 0.10 µM, NPTG and PTG, respectively) (Figure 6C). The oxaliplatin/gemcitabine mix differently affected the HCT116 viability with respect to the PTG complex. Thus, the cytotoxic effects of the two concentrations of NPTG were compared to the effects of nanoparticles synthesized with the correspondent amount of oxaliplatin and gemcitabine combination (NP Pt + Gem, Figure 6D). As evidence, the nanoparticles loading with the combination of oxaliplatin and gemcitabine produced a more pronounced inhibition with respect to the NPTG. Altogether, these data highlighted that the nanoparticle formulations affect the cytotoxic profile of the PTG complex and speed up its effect. Nevertheless, it is interesting to notice, as in the case of free drugs, the slightly high activity of encapsulated oxaliplatin + gemcitabine mixture with respect to NPTG, which likely relies on kinetic reasons. Indeed, to exert the desired effects, PTG undergoes a reduction and subsequent release of all the active species that, only after the activation stage, may exert their anticancer activity.

## 4. Conclusions

With the aim of addressing the challenges associated with platinum-based anticancer drugs, specifically the adverse side effects and the emergence of drug resistance, in this paper, we focused on combining well-established anticancer agents within a Pt(IV) scaffold. The design and synthesis of two Pt(IV) prodrugs, PTC and PTG, involved the integration of renowned anticancer agents, capecitabine and gemcitabine, into the axial positions of the oxaliplatin-based Pt(IV) scaffold. This promising strategy aims to consolidate the therapeutic benefits of these agents into a single molecule, potentially alleviating side effects and circumventing drug resistance observed in the established treatments. Significant insights into the anticancer activity of PTC and PTG were gained through the evaluation of the growth inhibition potential on HCT116 colon cancer cells. In the case of PTC, the IC_50_ value turned out to not be improved with respect to oxaliplatin (the reference compound for colorectal cancer). On the contrary, potent advantages were found for PTG, revealing interesting growth inhibition with an IC_50_ value of 0.49 ± 0.04 µM after 72 h of incubation. In general, the observed induction of cell cycle arrest and apoptosis by the complexes underscores their multifaceted impact on cancer cell proliferation, suggesting diverse mechanistic pathways. Importantly, superior activity was exhibited by PTG, emphasizing its potential as an effective anticancer agent. Based on these premises, for further studies, we focused on PTG only. Reactivity studies with PTG provided valuable insights, confirming the formation of active Pt(II) species, namely oxaliplatin and gemcitabine, under reducing conditions typical of cancer cells. Cyclic voltammetry results supported the notion of reductive activation, elucidating the electrochemical behavior of PTG within a biologically relevant potential range. Finally, the encapsulation of PTG in PLGA-PEG nanoparticles (NPTG) further demonstrated its accelerated cytotoxicity, indicating the potential of nanoparticle delivery systems to enhance therapeutic outcomes. In conclusion, this study unveils the promising use of Pt(IV) prodrugs as a valuable strategy in cancer therapy. The synergistic combination of established chemotherapeutic agents within a Pt(IV) scaffold, coupled with the potential benefits of nanoparticle delivery systems (that ensure the retention of and improvement in pharmacological profile with respect to the free drug), paves the way for further exploration and development of more effective and tolerable anticancer treatments.

## Figures and Tables

**Table 1:** IC_50_ Values (µM) of different compounds on HCT116 cells.

Compound	IC_50_ (µM) ± SEM
Oxaliplatin	29 ± 9
Gemcitabine	0.53 ± 0.05
Capecitabine	>1000
PTG	0.49 ± 0.04
PTC	50 ± 8

**Table 2:** IC_50_ Values (µM ± SEM) of NP compounds on HCT116 cells after 24, 48, and 72 h of incubation.

Compound	IC_50_ 24 h	IC_50_ 48 h	IC_50_ 72 h
PTG	2.3 ± 0.2	1.7 ± 0.2	0.50 ± 0.10
NPTG	1.37 ± 0.19	1.21 ± 0.03	0.44 ± 0.08

## Data Availability

The original contributions presented in the study are included in the article/supplementary material, further inquiries can be directed to the corresponding author/s.

## Supplementary Materials

The following supporting information can be downloaded at: https://www.mdpi.com/article/10.3390/pharmaceutics16020278/s1, Figures S1–S3: Cellular experiments; Figures S4–S8: ESI-MS spectra; Figures S9 and S10: ^1^HNMR spectra.
