# Supplementary material for: Oxaliplatin(IV) Prodrugs Functionalized with Gemcitabine and Capecitabine Induce Blockage of Colorectal Cancer Cell Growth—An Investigation of the Activation Mechanism and Their Nanoformulation
Source: Pharmaceutics. 2024 Feb 16;16(2):278. doi: 10.3390/pharmaceutics16020278 (PMC10892879; doi:10.3390/pharmaceutics16020278)
Supplement: Supplementary file 1 [file pharmaceutics-16-00278-s001.zip › pharmaceutics-2867793-supplementary.pdf]

Supplementary Materials

Oxaliplatin(IV) Prodrugs Functionalized with Gemcitabine and Capecitabine Induce Blockage of Colorectal Cancer Cell Growth—An Investigation of the Activation Mechanism and Their Nanoformulation

Carlo Marotta [1,†], Damiano Cirri [1,*,†], Ioannis Kanavos [2], Luisa Ronga [2], Ryszard Lobinski [2], Tiziana Funaioli [1], Chiara Giacomelli [3], Elisabetta Barresi [3], Maria Letizia Trincavelli [3], Tiziano Marzo [3] and Alessandro Pratesi [1,*]

[1] Department of Chemistry and Industrial Chemistry, University of Pisa, 56124 Pisa, Italy; carlo.marotta@phd.unipi.it (C.M.); tiziana.funaioli@unipi.it (T.F.)

[2] Institute of Analytical and Physical Chemistry for the Environment and Materials (IPREM-UMR 5254),
Pau University, E2S UPPA, CNRS, 64053 Pau, France; i.kanavos@univ-pau.fr (I.K.); luisa.ronga@univ-pau.fr (L.R.); ryszard.lobinski@univ-pau.fr (R.L.)

[3] Department of Pharmacy, University of Pisa, 56126 Pisa, Italy; chiara.giacomelli@unipi.it (C.G.); elisabetta.barresi@unipi.it (E.B.); maria.trincavelli@unipi.it (M.L.T.); tiziano.marzo@unipi.it (T.M.)

* Correspondence: damiano.cirri@unipi.it (D.C.); alessandro.pratesi@unipi.it (A.P.)

† These authors contributed equally to this work.

Contents:

1. Cellular experiments 2
2. ESI mass spectra 5
3. Comparison of theoretical and experimental isotopic patterns 6
4. ^{1}HNMR spectra 8

1. Cellular experiments

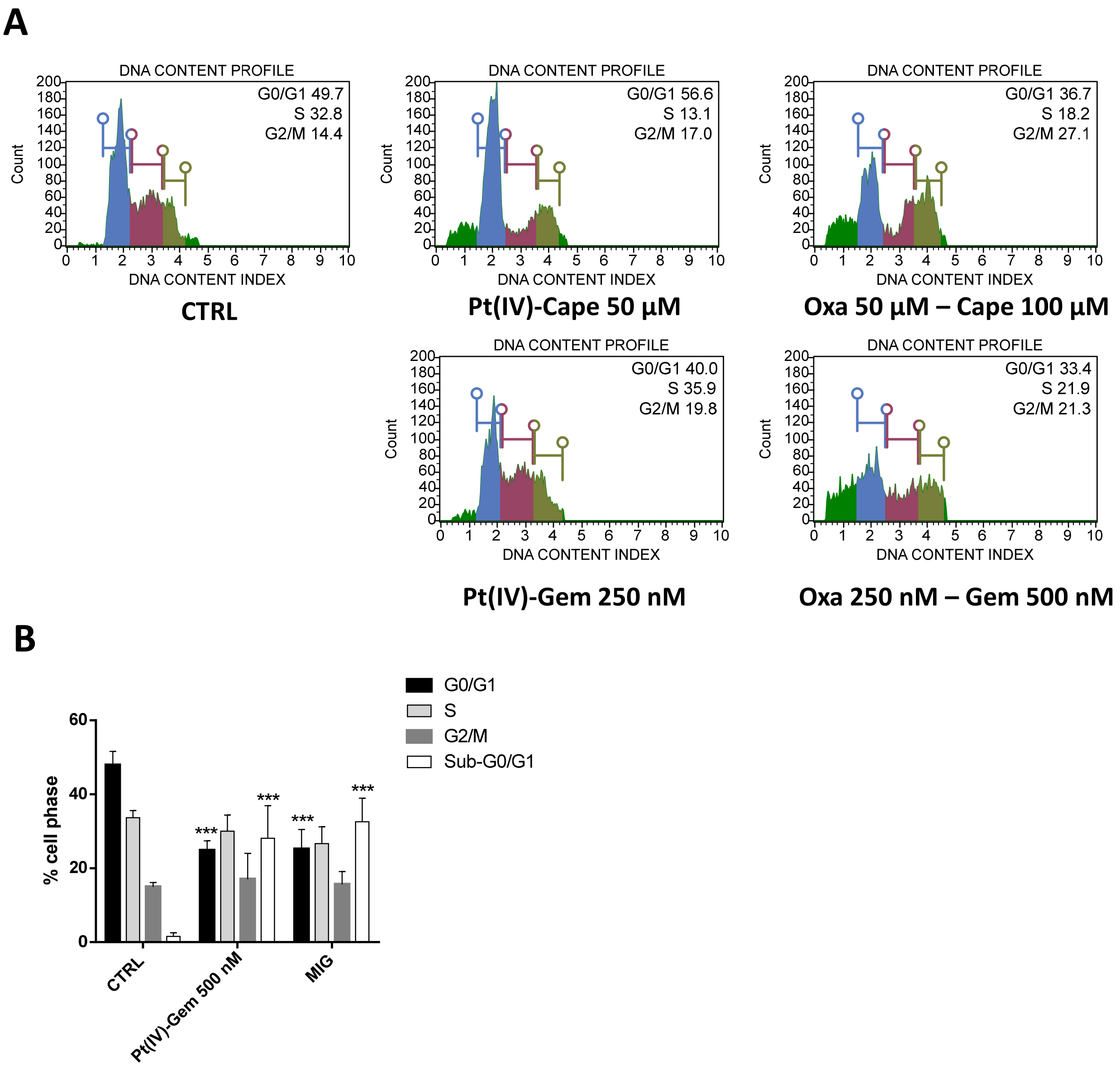

Figure S1. HCT116 cells were treated as reported for 48 h. After incubation time, the cell cycle analyses were performed. (A) Representative cell cycle histograms of untreated and treated cells were reported. (B) Data are expressed as the percentage of cells in the different phases (sub-G0/G1, G0/G1, S, or G2/M) versus total cell number. Data represent the mean ± SD of three different experiments. The statistical significance of the data was determined with a one-way ANOVA with Bonferroni post-test: *$p<0.05$, **$p<0.01$, ***$p<0.001$ vs respective phase of the CTRL

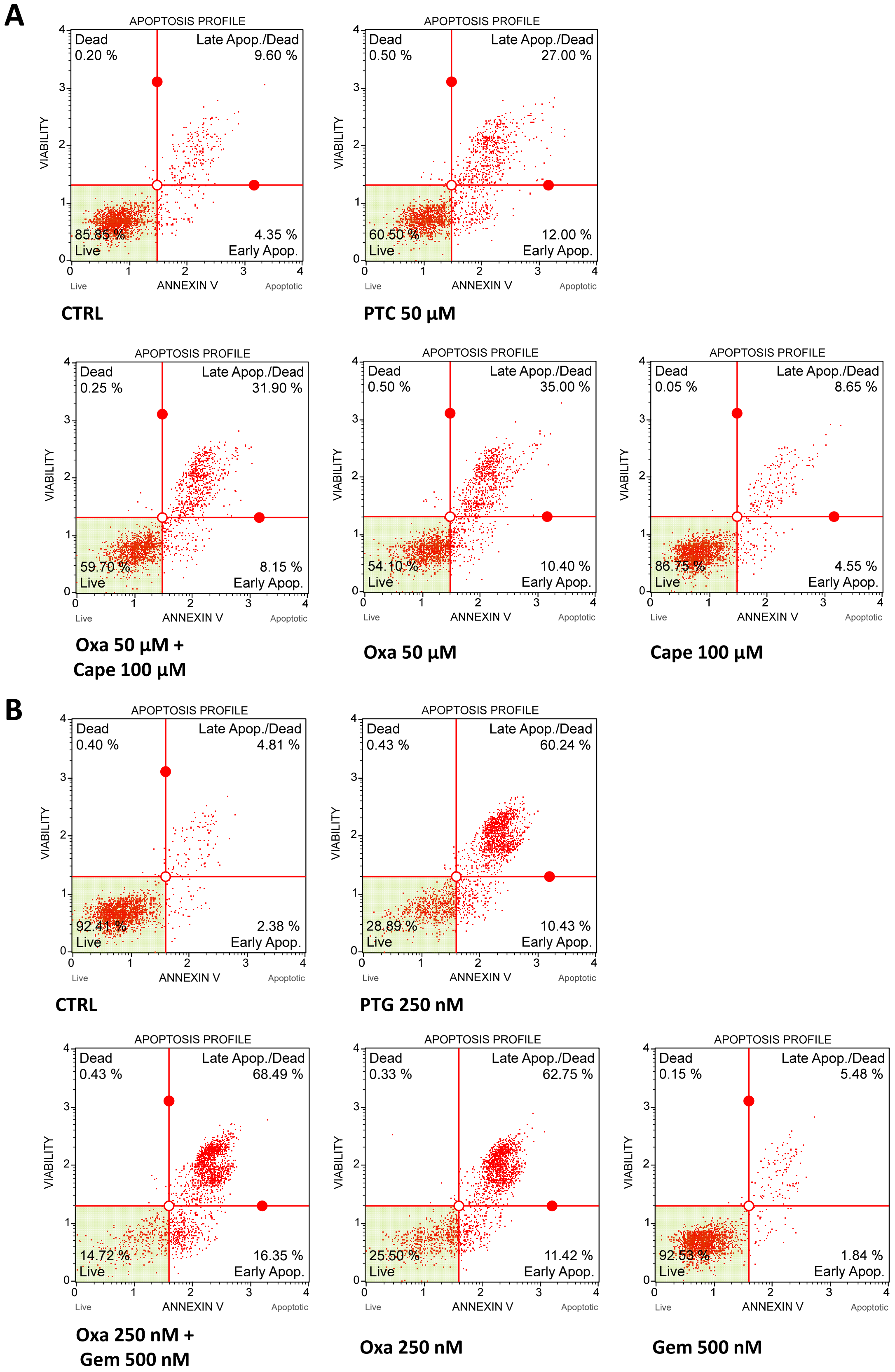

Figure S2. HCT116 cells were treated as reported for 48 h. After incubation time, the quantification of apoptotic cells was performed. Representative histograms of untreated and treated with PTC (A) and PTG (B) cells were reported.

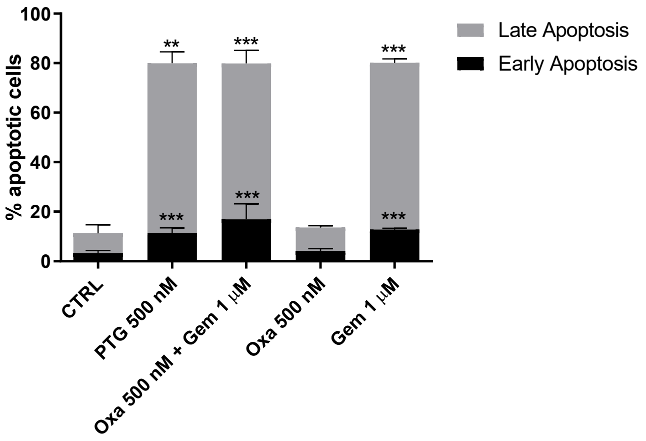

Figure S3. HCT116 cells were treated as reported for 48 h. After incubation time, the quantification of apoptotic cells was performed. The phosphatydilserine externalization was quantified using Annexin V staining. The data are expressed as the percentage of apoptotic cells (Early-apoptotic in black, late-apoptotic in grey) versus the total number of cells. Data represent the mean ± SD of three different experiments. The statistical significance of the data was determined with a one-way ANOVA with Bonferroni post-test: *p<0.05, **p<0.01, ***p<0.001 vs respective early or late apoptosis of the CTRL.

2. ESI mass spectra

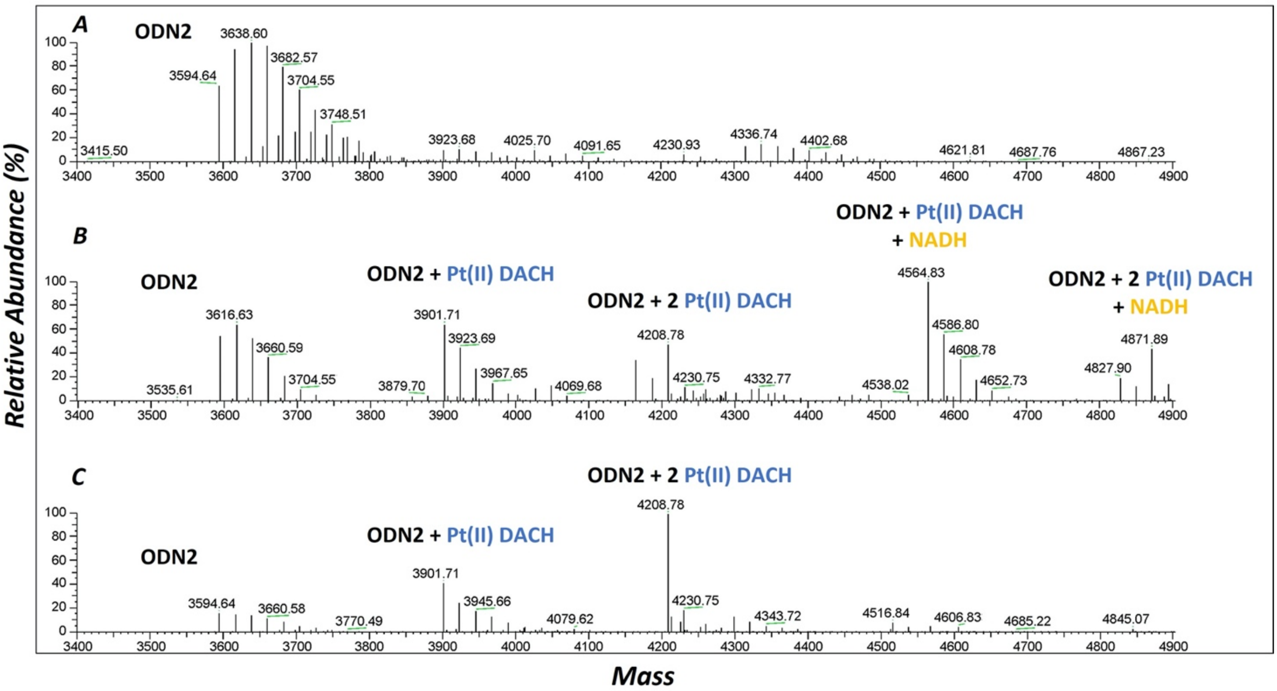

Figure S4. Deconvoluted ESI mass spectra of **(A)** ODN2 (10^{-4} M) incubated at 37 °C for 48 h with Pt(IV)-Gem (1:3 ODN2 to Pt(IV)-Gem ratio) in 2 mM ammonium acetate buffer pH=7.0, **(B)** ODN2 (10^{-4} M) incubated at 37 °C for 48 h with Pt(IV)-Gem (1:3 ODN2 to Pt(IV)-Gem ratio) in 2 mM ammonium acetate buffer pH=7.0 in the presence of NADH (4:1 NADH to Pt(IV)-Gem ratio) and FMN (1:4 FMN to Pt(IV)-Gem ratio), **(C)** ODN2 (10^{-4} M) incubated at 37 °C for 48 h with oxaliplatin (1:3 ODN2 to oxaliplatin ratio) in 2 mM ammonium acetate buffer pH=7.0

3. Comparison of theoretical and experimental isotopic patterns

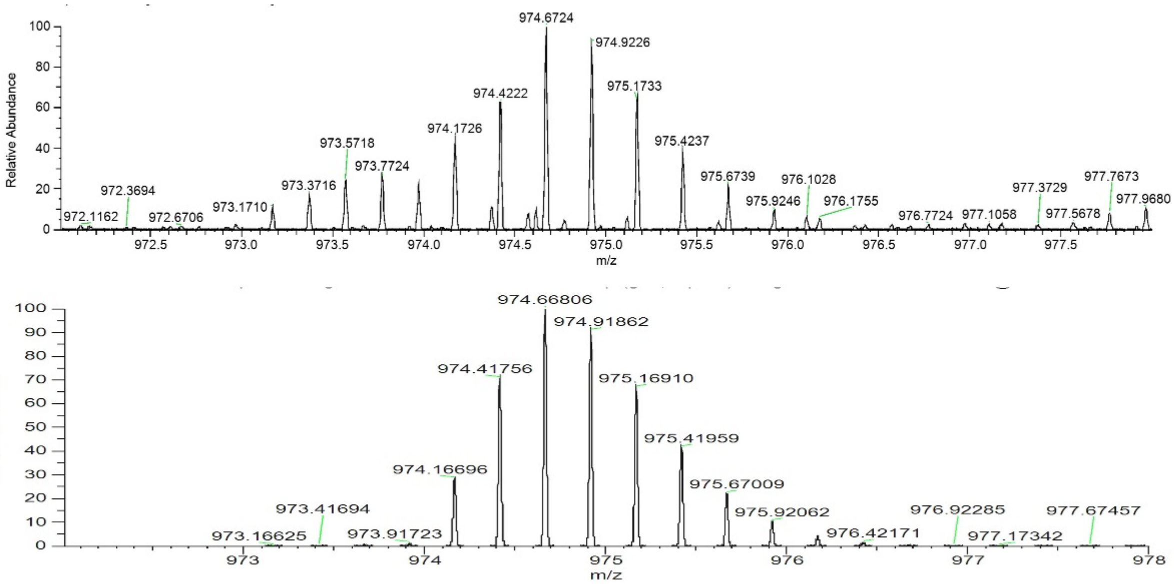

Figure S5. ODN2 + Pt(II) DACH, 974.6724 ion (*m/z*) ($C_{122}H_{161}N_{42}O_{72}P_{11}Pt_1$): comparison of experimental (top) and theoretical (bottom) (calculated as $[M-4H]^{4-}$) isotopic patterns.

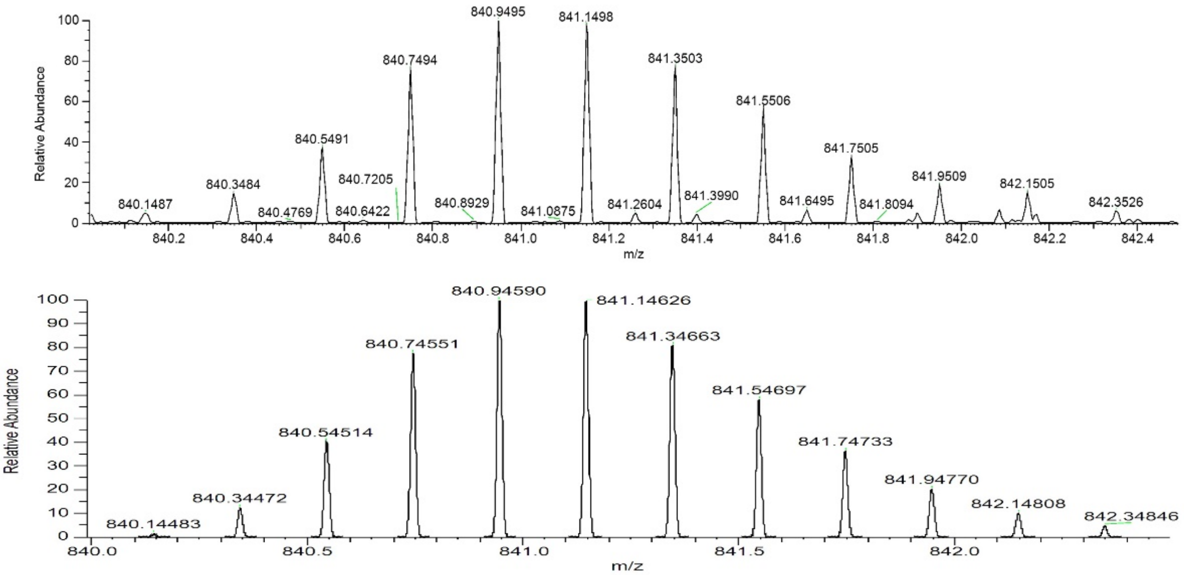

Figure S6. ODN2 + 2 Pt(II) DACH, 840.9495 ion (*m/z*) ($C_{128}H_{173}N_{44}O_{72}P_{11}Pt_2$): comparison of experimental and theoretical (calculated as $[M-5H]^{5-}$) isotopic patterns.

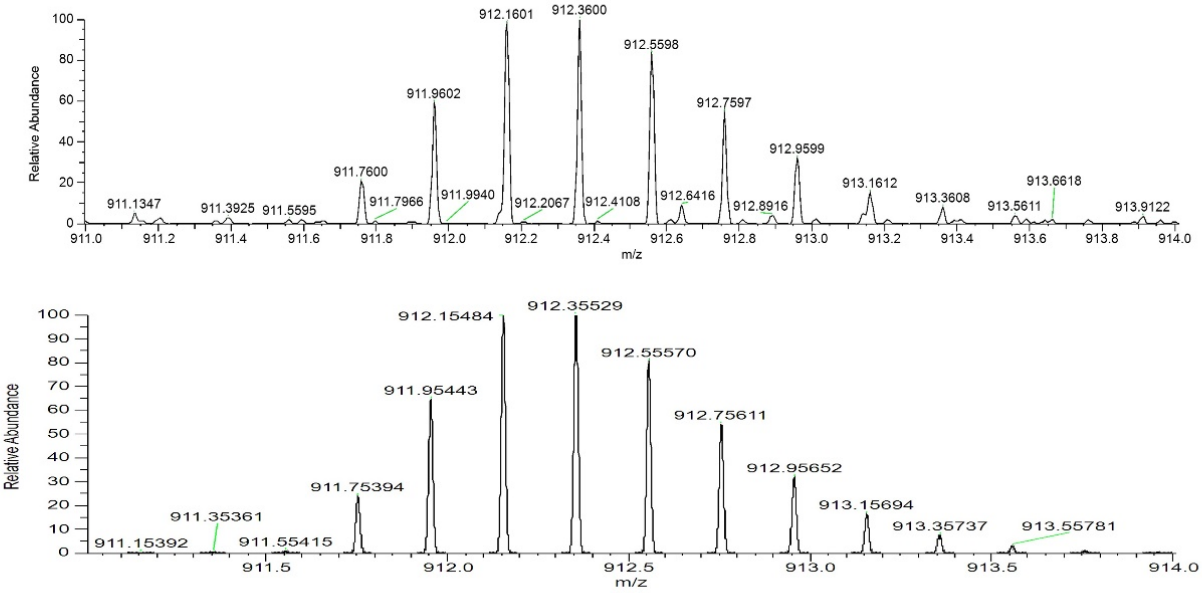

Figure S7. ODN2 + Pt(II) DACH + NADH, 912.3600 ion (*m/z*) ($C_{143}H_{183}N_{49}O_{86}P_{13}Pt$): comparison of experimental and theoretical (calculated as $[M\text{-}5H]^{5-}$) isotopic patterns.

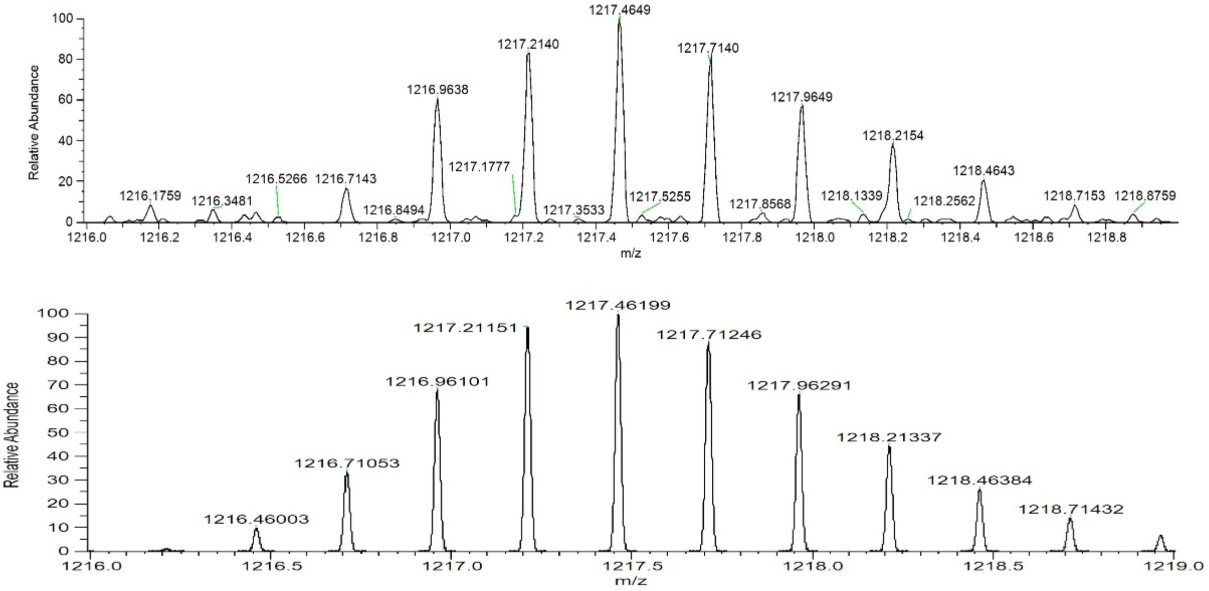

Figure S8. ODN2 + 2 Pt(II) DACH + NADH, 1217.4649 ion (*m/z*) ($C_{149}H_{196}N_{51}O_{86}P_{13}Pt_2$): comparison of experimental and theoretical (calculated as $[M\text{-}4H]^{4-}$) isotopic patterns.

4. ^{1}HNMR spectra

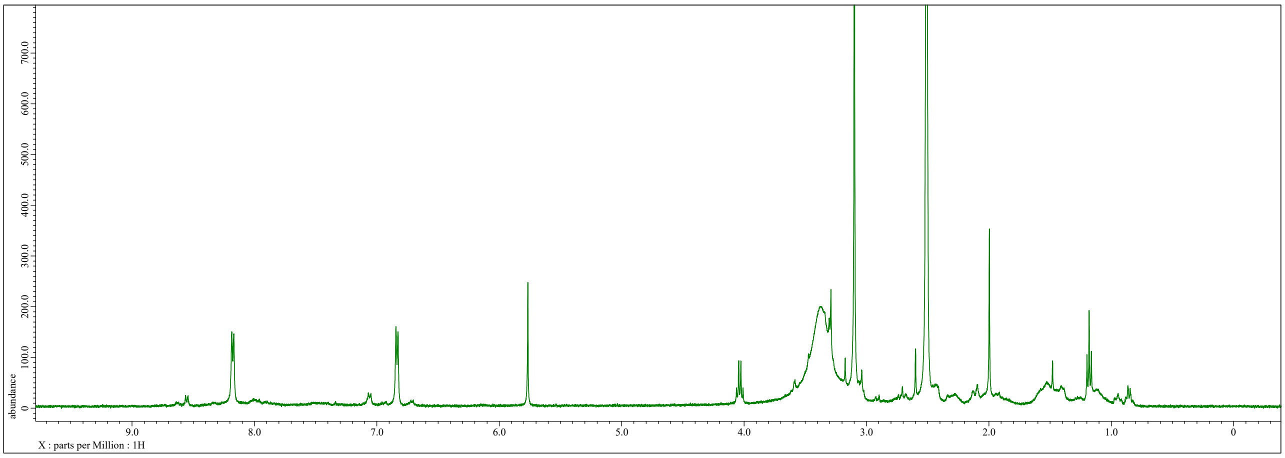

Figure S9. ^{1}HNMR spectrum of PTC.

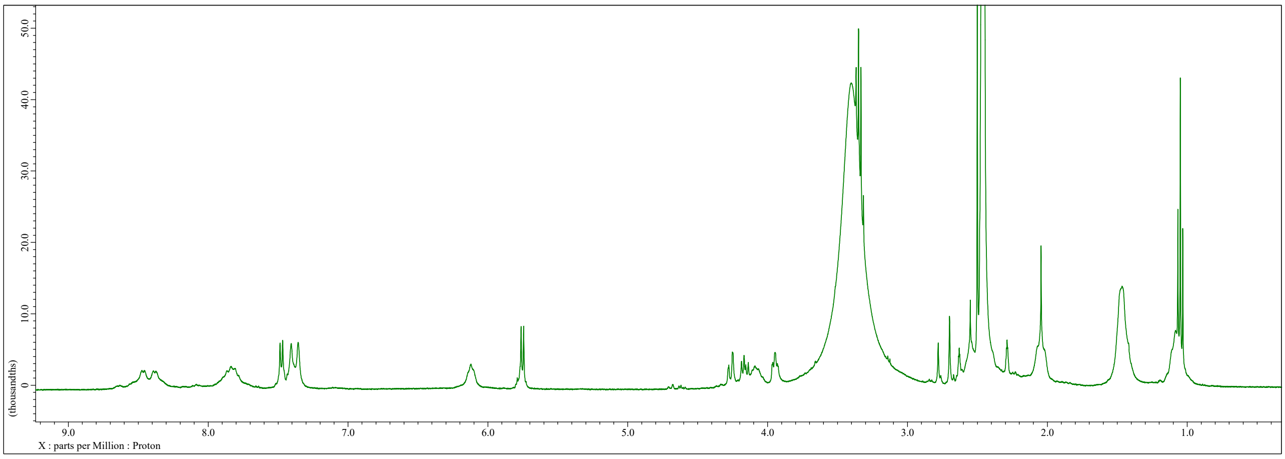

Figure S10. ^{1}HNMR spectrum of PTG.
